# Determinant factors of leprosy-related disability; comparison of acceleration failure time and parametric shared frailty models

**DOI:** 10.1371/journal.pone.0271883

**Published:** 2023-04-03

**Authors:** Bezanesh Melese Masresha, Kasim Mohammed Yesuf, Yikeber Abebaw Moyehodie, Hailegebrael Birhan Biresaw, Solomon Sisay Mulugeta, Gedam Derbew Addisia

**Affiliations:** 1 Debre Tabor University, Debre Tabor, Amhara, Ethiopia; 2 St. Paul’s Hospital Millennium Medical College School of Public Health, Addis Ababa, Ethiopia; Public Library of Science, UNITED KINGDOM

## Abstract

**Background:**

Leprosy is an illness persisting for a long time or constantly recurring brought about by *Mycobacterium leprae*. The collusion of the causing agent with Schwann cells leads to incapable of being changed loss of fringe nerve tissue; followed by incapacity and that is not restricted to actual powerlessness yet additionally makes a negative picture, prompting segregation and social disgrace against the altered people also, their families.

**Methods:**

The analysis of this study comprises 205 samples of patients at All African TB and Leprosy Rehabilitation and Training Centre from January 2015 up to December 2019 G.C who were taking medication for leprosy and who possess all necessary data. Territorial conditions in the region of the patients were utilized as a clustering impact in all frailty models. Acceleration failure time models and parametric shared frailty models with Weibull and log-strategic patterns were utilized to dissect hazard factors related to disability ensued by leprosy. All fitted models were looked at by utilizing AIC.

**Results:**

From that of 205, 69(33.7%) experienced at least one kind of disability grade during treatment taking. In light of AIC, log-logistic-gamma shared frailty model was the final best fitting model and also there was considerable variation among patients. The final model showed the age of patients, symptom duration, treatment category of patients, and sensory loss were found to be the most significant determinants of leprosy disability.

**Conclusion:**

In this investigation, there is proof of heterogeneity at the group level and disability was related to the age of patients, symptom duration, treatment category of patient, what’s more, sensory loss subsequently, uncommon consideration ought to be given to these huge indicators, which eventually diminish the event of disability. To lessen the patient-related postponement, the program should lay more noteworthy accentuation on bringing issues to light in the local area by zeroing in on key messages like indications, inability result of the late discovery, accessibility of free treatment what’s more, accessibility of disease care in general wellbeing office.

## Introduction

Leprosy is an illness persisting for a long time or constantly recurring brought about by *Mycobacterium leprae*. The causative agent affects peripheral nerves and causes damage by binding to Schwann cells which are important for conducting nerve impulses [1]. The interaction of *M. leprae* with Schwann cells causes irreversible loss of peripheral nerve tissue followed by disabilities. In some cases, nerve damage occurs without skin lesions which makes the diagnosis difficult leading to further damage. Leprosy is considered an important public health problem due to its morbidity and socioeconomic impact, both of which are consequences of complications (e.g., physical disability and deformities) that develop during the clinical outcome of the disease [2]. The damage is not limited to the physical inability but also creates a negative image leading to discrimination and social stigma against the affected individuals and their families [3].

Physical disability can occur before leprosy diagnosis, during treatment, and post-release from treatment [4]. About 15% of the world’s population has some form of disability [5]. The Global Leprosy scheme 2016–2020 spotlights early case recognition before apparent incapacities happen. Target recognition among higher danger bunches through leading efforts in profoundly endemic regions or networks; and further developing inclusion and access for underestimated populaces. This will bring about prior recognition and decrease of patients with gradeII disability (G2D) at the time of examination. The objective of G2D rate is less than one for every million populace [6].

According to the Global Leprosy (Hansen’s disease) Strategy 2021–2030 in the absence of verifiable data, it is estimated that 3–4 million people are living with visible impairments or deformities due to leprosy. Because both the label of leprosy and the disability it causes result in social exclusion in many communities, the number of people experiencing leprosy-related stigma is likely to be even greater [7]. According to official reports received from the World Health Organization, 214,783 new cases of leprosy were detected in 2016. India was one of the countries with the highest leprosy burden with more than 135,000 new leprosy patients being detected every year, including 5,245(3.9%) new leprosy patients with a visible disability: grade two disability (G2D). In the year 2016, India reported 63% of the world’s new leprosy cases; about 40% of the world’s new G2D among new leprosy cases. India reported an increasing trend of new cases with G2D in the period 2008- 2015 from 3.1% to 4.6%. A total of 3,848 new cases with G2D were detected by contact examination in the leprosy case detection campaign from April 2017 to March 2018 [8].

There were 202,185 new leprosy cases registered globally in 2019, according to official figures from 161 countries in the 6 WHO Regions. Of them, 14,981 were children below 15 years and the new case detection rate among the child population was recorded at 7.9 per million child population. Based on 178,371 cases at the end of 2019, the prevalence corresponds to 25.9 per million population. Among the new cases, 10,813 new cases were detected with gradeII disabilities (G2D) and the G2D rate was recorded at 1.4 per million population [9].

In 2020, 127 countries provided information on leprosy. The registered prevalence of leprosy (the number of cases on treatment at the end of 2020) was 129,192, with a rate of 16.6 per million population. Globally, 127,396 new cases were reported, for a case detection rate of 16.4 per million population. Both figures were much lower than in previous years, with a 27.7% reduction in registered prevalence and a 37.1% reduction in new cases as compared with 2019. The highest proportions of both cases registered for treatment (61.1%) and new cases detected (66.6%) were in SEAR. Brazil, India and, Indonesia reported 72.5% of registered cases and 74.0% of new cases detected in 2020 [10].

Despite achieving the elimination goal in 1999, Ethiopia still has the second-highest leprosy disease burden in Sub-Saharan Africa (SSA) [11]. By the report of Glra-Ethiopia Annual Master Report (2015) the national tuberculosis and leprosy control program received 3,500 to 4,000 new leprosy cases between 2013 and 2015. According to WHO, the country reported 3,201 new leprosy patients in 2019, with 12.8% of them having a gradeII disability as reported by WHO [12]. Among regions, 2,046 leprosy cases (49%) were reported from Oromia followed by the Amhara region with 1,409 cases (34%) and the SNNPR region with 348 cases (8%). These three main regions in Ethiopia constituted 91% of all cases reported [13].

While an assortment of investigations have been done on leprosy and components that lead to the improvement of actual disabilities and distortions in Ethiopia, no examination reported on the space of leprosy disability by utilizing parametric shared frailty models aside from the studies were directed by utilizing logistic regression. Logistic regression doesn’t account for the editing perceptions, i.e., doesn’t hold for time-to-event information; in any case, endurance investigation is more impressive than a Logistic structure that takes censoring into consideration. Accordingly, this research gave an augmentation of Cox PH model which is the frailty model, taking into account any additional heterogeneity present in the information by utilizing a shared frailty model to examine the determinants related to disability among patients enlisted in All African TB and Leprosy Rehabilitation and Training Center, Addis Ababa, Ethiopia. The frailty term thinks to the circumstance where a portion of the patients might be presented to the danger of disability higher than the others. Thus, knowledge of the main risk factors for the development of physical disability is important for disability prevention programs because this knowledge provides access to important predictors of better surveillance [14].

## Data and methodology

### Source of data

All medical records of registered leprosy patients at ALERT Centre from January 2015 up to December 2019 G.C in Addis Ababa, Ethiopia, were retrospectively reviewed by medical professionals. The data consists of patients that admitted to the center with leprosy cases. The total numbers of leprosy patients registered during this study were 807. Of those the aggregate of 807 the patients enlisted, only 205 leprosy patients whose card had full information and hence are included in this study.

#### Ethical approval and consent to participate

The data used in the current investigation was collected previously by the health staff for treatment purposes/for diagnosis of leprosy and to start follow-up treatment. To use this previously collected data, an Ethical approval certificate has been obtained from the ethical clearance review committee of the College of Natural and Computational Science, University of Gondar. University of Gondar, Ethiopia, with reference number: CNCS/10 627/05/19/2020. In data collection, there was no written or verbal consent from participants. The reason was the investigators did NOT get participants rather, secondary data were obtained in the patient’s chart. The Ethical approval committee approved the use of this secondary data for the current investigation. The data was de-identified by the investigators.

### Study design

A retrospective cohort study was carried out to retrieve relevant information from the medical records of leprosy patients registered from January 2015 up to December 2019 at the ALERT center, Addis Ababa, Ethiopia.

### Inclusion and exclusion criteria

This study includes leprosy patients who start the medication with disability grade G0D or G1D registered for treatment with MDT at any time from January 2015 up to December 2019 at ALERT Center. All patients with incomplete data, and those who were transferred to other locations during treatment were excluded. Since G2D is the final disability grade patients with this category will never experience the event of the study that was a new occurrence of some disability grade (G1D or G2D) for the patient during treatment and therefore those leprosy patients with a history of gradeII disability (G2D) before starting the treatment during the study period were excluded. Thus, by considering such reasons and the availability of the required data in the hospital, in this study, the appropriate sample size was taken all patients based on the eligibility criteria.

The patients were categorized as having G0D, G1D and G2D as per the WHO disability grading system. A patient manifesting no sensory or motor nerve function impairment or loss of vision due to leprosy was considered as having a G0D disability. G1D disability was diagnosed whenever a patient presented with sensory nerve function impairment affecting hands or feet without motor nerve function impairment affecting these sites (due to leprosy) or visual impairment better than 6/60 due to leprosy. G2D disability was diagnosed when there was motor nerve function impairment or visible deformity of hands or feet or visual impairment <6/60 due to leprosy [15].

### Variables in the study

#### Dependent variable

The outcome variable was the endurance season of patients with leprosy from the date of enlistment to the treatment care until the finish of the examination (in months). Disability was considered as the event of the study and the reaction time was the point at which the patient has some disability (G1D or G2D). The patient was considered as censored in case they were misfortune to follow-up, if they were cured before experiencing a new disability grade and if they do not experience a new disability up to the study end.

As described the dependent variable was the survival time of patients with leprosy from the date of enrollment for the treatment care till the end of the study, the occurrence of some disability grade was considered as the event of the study and the response time was the time at which the patient has a new disability upgrading, but the disability grade experienced by the leprosy patient can be either G1D or G2D. At the beginning of treatment, patients may have G0D, G1D, or G2D, but we considered the new disability grade that occurred during treatment taking as an event only for patients with G0D and G1D. There is no any more disability upgrading during treatment for leprosy patients with G2D because they already have experienced the final disability grade before starting the medication.

#### Independent variables

The determinant factors considered in this study were described as individual qualities furthermore, clinical components. A synopsis of the factors considered in this investigation are introduced in Table 1.

**Table 1:** Depiction of covariates that were incorporated for the study.

No.	variable	categories
1.	Sex	1 = male, 2 = female
2.	Age	Continues
3.	Region	1 = Addis Ababa, 2 = Amhara 3 = Oromiya, 4 = SNNPR 5 = Others
4.	Starting disability grade	0 = G0D 1 = G1D
5.	Contact (Exposure) history	1 = No (patient Does not know by what they have developed the disease) 2 = By Contact out of the family 3 = Family
6.	First lesion body part	1 = Hand, 2 = Leg 3 = Hand and Leg(HL), 4 = Face
7.	Sensory loss	1 = Moderate, 2 = Marked 3 = Absent
8.	Symptom duration (Duration)	Continues
9.	Distribution of skin lesion	1 = Symmetric, 2 = Part Symmetric, 3 = Asymmetric
10.	Type of leprosy	0 = Pauci Bacillary, 1 = Multi Bacillary
11.	Treatment category (Category)	1 = Relapse, 2 = Defaulter, 3 = New
12.	Smear result	0 = positive, 1 = negative
13.	Thickened nerve	1 = Yes, 2 = No

### Method of data analysis

#### Non-parametric methods

The Kaplan-Meier estimator, also known as the product-limit estimator, was presented by [16]. It gives a simple and quick estimate of the survival function in the presence of censoring. The Kaplan-Meier estimator is denoted as $\hat{S}(t)$ and given by:

$$\begin{array}{c} \hat{S}\left(t\right)=\prod_{j:\tau_{j}<t}^{}\left[1-\frac{d_{j}}{n_{j}}\right], \end{array}$$ (1)

where *d*_*j*_ is the number of disabled at *τ*_*j*_, *n*_*j*_ is the number of individuals “at risk” right before the *j*^*th*^ disability time(every one disabled or censored at or after that time).

### Accelerated Failure Time (AFT) model

In survival data analysis, survival models can also be used in addition to the hazards model. One advantage of such models is that the proportionality assumption of the hazards is not required. The parametric survival models work analogous to the multiple linear regression of the logarithm of survival time on explanatory variables. Such survival models are termed as a parametric accelerated failure time models or simply AFT models. Because these models work on survival, the complementary concept of hazard, the sign of the regression coefficients in an AFT model will be opposite to those in PH models [17].

Most usually utilized parametric AFT models are Exponential, Weibull, Log-Logistic, Log-normal furthermore, Generalized Gamma. Exponential and Weibull parametric models can work both in PH metric and in AFT measurement. These models are similarly proper seen in one or the other measurement. What’s more, one can change relapse coefficients processed in PH metric into the relapse coefficient in AFT measurement or the other way around for Exponential and Weibull parametric survival models. Under Toward the back models direct impact of the informative factors estimated on the survival time all things considered of risk. This trademark takes into account a simpler understanding of the outcomes in light of the fact that the boundaries measure the impact of the reporter covariate on the mean endurance time. The individuals from the AFT model considered in this study are the Weibull AFT and log-logistic AFT models.

#### Weibull accelerated failure time model

The Weibull distribution (counting the Exponential distribution as an uncommon case) can be defined as an AFT model and they are the solitary group of dispersions to have this property. The Weibull distribution is the truly adaptable model for time-to-event information. It has a danger rate that is droning expanding, diminishing, or steady [17]. The Accelerated Failure Time (AFT) portrayal of the survival and hazard function of the Weibull model with scale boundary and shape boundary is given by:

$$\begin{array}{c} f\left(t\right)=\frac{\alpha }{\lambda }{\left[\frac{t}{\lambda }\right]}^{\alpha -1}\text{exp}(-{\left[\frac{t}{\lambda }\right]}^{\alpha }) \end{array}$$ (2)

0≤t<∞ where, λ and *α* are the scale and shape parameters respectively.

And the baseline hazard of this model for the i^*th*^ subject is

$$\begin{array}{c} \lambda \left(t\right)=\frac{\alpha }{\lambda }{\left[\frac{t}{\lambda }\right]}^{\alpha -1} \end{array}$$ (3)

#### Log-logistic accelerated failure time model

The log-logistic distribution has a genuinely adaptable practical structure, it is one of the parametric survival models in which the peril rate might be diminishing, expanding, just as protuberance formed that is at first increments and afterward diminishes. In situations where one goes over to censored data, utilizing log-logistic appropriation is numerically more favorable than other conveyances. The log-logistic model has two boundaries. Then, at that point, the AFT portrayal of the log-logistic survival function is given by:

$$\begin{array}{c} f_{0}\left(t\right)=\frac{\lambda^{k}{\left[kt\right]}^{k-1}}{{\left[1+{\left(t\lambda \right)}^{k}\right]}^{2}} \end{array}$$ (4)

$$\begin{array}{c} S_{0}\left(t\right)=\frac{1}{1+{\left[\lambda t\right]}^{k}} \end{array}$$ (5)

And the associated hazard function for the i^*th*^ individual also as follow:

$$\begin{array}{c} h_{0}\left(t\right)=\frac{\lambda^{k}{\left[kt\right]}^{k-1}}{1+{\left(t\lambda \right)}^{k}} \end{array}$$ (6)

### Shared frailty model

The shared frailty model is a conditional independence model in which frailty is common to all subjects in a cluster. This model is responsible for creating a dependence between event times. It is also known as a mixture model because the frailties in each cluster are assumed to be random. It assumes that, given the frailty, all event times in a cluster are independent. The shared frailty model was introduced by [18] without using the motion of frailty and extensively studied in [19]. Suppose we have j observations and i subgroups. Each subgroup consists of *n*_*i*_ observations and

$$\begin{array}{c} \sum_{i=1}^{n}n_{i}=n \end{array}$$ (7)

Where *n* is the total sample size. The hazard rate for the *j*^*th*^ individual in the *i*^*th*^ subgroup is given by

$$\begin{array}{c} h_{ij}\left(t\right)=h_{0}\left(t\right)u_{i}\text{exp}\left(z_{ij}^{t}\beta \right),i=1,.........G,j=1,2,......n \end{array}$$ (8)

where *u*_*i*_ are frailty terms for subgroups and their distribution is again assumed to be independent with a mean of 0 and a variance of 1. If the number of subjects *n*_*i*_ is 1 for all groups, the univariate frailty model is obtained [20]; otherwise, the model is called the shared frailty model [19] because all subjects in the same cluster share the same frailty value.

#### Shared gamma distribution

The Gamma frailty model belongs to the power variance function family [21] and can be expressed in terms of its Laplace transform from which properties such as mean and variance are easily derived [22]. From a computational and analytical point of view, it fits very well with failure data. It is widely used due to its mathematical tractability [20]. Assuming a two-parameter gamma density with *σ* >0 and *γ* >0 as shape and scale parameters respectively. The density function is given by

$$\begin{array}{c} f_{z}\left(z_{i}\right)=\frac{\gamma^{\sigma }z_{i}^{\sigma -1}\text{exp}\left(-\gamma z_{i}\right)}{\Gamma (\sigma )} \end{array}$$ (9)

with *σ* > 0 and *γ* > 0 and where Γ(.) is the Gamma function. The corresponding Laplace transformation is

$$\begin{array}{c} L\left(s\right)=\gamma^{\sigma }{\left(s+\gamma \right)}^{-\sigma } \end{array}$$ (10)

In gamma frailty model, a restriction *γ* = *σ* is used, which results an expectation of 1. The variance of the frailty variable is then 1. Assuming that the frailty term *z*_*i*_ is a gamma with E(Z) = 1 and var(Z) = *θ*, then *γ*=*σ* = $\frac{1}{\theta }$ [23]. The distribution function of the frailty term *z*_*i*_ is therefore a one-parameter gamma distribution given by

$$\begin{array}{c} f_{z}\left(z_{i}\right)=\frac{z_{i}^{(\frac{1}{\theta })-1}\text{exp}\left(-\frac{z_{i}}{\theta }\right)}{\Gamma \left(\frac{1}{\theta }\right)\theta^{\frac{1}{\theta }}} \end{array}$$ (11)

Where Γ(.) is the gamma function. It corresponds to a Gamma distribution Gam(*μ*, *θ*) with *μ* fixed to 1 for identifiability and its variance is *θ*.

#### Positive stable shared frailty distribution

The positive stable (PS) model [19] is a useful alternative to a gamma distribution, because it has the attractive feature that the predictive hazard ratio decreases to 1 over time [24]. The property is observed in familial associations of the age of onset of disease with etiologic heterogeneity, where genetic cases occur early and long-term survivors are weakly correlated. The gamma model has predictive hazard ratios which are time invariant and may not be suitable for these patterns of failures [25]. The probability density function (pdf) of a positive stable distribution with two parameters *α* and *σ*, restricting the parameters (*α*=*σ*) to solve the non-identifiable problem is given by: [19].

$$\begin{array}{c} f\left(z\right)=\frac{1}{n}\sum_{k=1}^{\infty }\frac{\Gamma (k\alpha +1)}{k!}{\left(-\frac{1}{2}\right)}^{\alpha k+1}\text{sin}(\alpha k\pi )\\ \text{z}>0,0<\alpha <1,\delta >0 \end{array}$$ (12)

#### Inverse Gaussian shared frailty distribution

Inverse Gaussian frailties generate stronger dependence at mid-time. As an alternative to the gamma distribution, the inverse Gaussian distribution was introduced by [21] and has been used by [26]. Similar to the gamma frailty model, simple closed-form expressions exist for the unconditional survival and hazard functions, this makes the model attractive. The probability density function of an inverse Gaussian shared distributed random variable with parameter *θ* > 0 is given by:

$$\begin{array}{c} f_{z}\left(z_{i}\right)={\left(\frac{1}{2\pi \theta }\right)}^{\frac{1}{2z_{i}}-3/2\text{exp}(\frac{-{\left(z_{i}-1\right)}^{2}}{2\theta z_{i}})}\;\\ z>0,\theta >0 \end{array}$$ (13)

For identifiability, we assume z has an expected value equal to one and variance *θ*.

### Baseline hazard distribution

#### (i). Baseline Weibull distribution

The Weibull distribution is more general distribution compared to the exponential distribution because when *α*=1, the Weibull distribution simplifies to an exponential distribution. The Weibull model can fit a range of survival data because of its flexibility. When *α*=1, the shape parameter is set to one, the failure rate is a constant. When *α* >1, the failure rate is increasing and when *α* <1, we have a decreasing failure rate. Thus, the Weibull probability model can be used to model the survival times of populations whose hazard rate is assumed to be decreasing, increasing or constant. The probability density function of the Weibull distribution is given by:

$$\begin{array}{c} f\left(t\right)=\frac{\alpha }{\lambda }{\left[\frac{t}{\lambda }\right]}^{\alpha }\text{exp}(-{\left[\frac{t}{\lambda }\right]}^{\alpha }) \end{array}$$ (14)

0≤t<∞ where λ and *α* are the scale and shape parameters, respectively. The cumulative density function denoted as F(t)is given as:

$$\begin{array}{c} F\left(t\right)=1-\text{exp}(-{\left[\frac{t}{\lambda }\right]}^{\alpha }) \end{array}$$ (15)

The survival function S(t) is given as:

$$\begin{array}{c} S\left(t\right)=\text{exp}(-{\left[\frac{t}{\lambda }\right]}^{\alpha }) \end{array}$$ (16)

It follows that the hazard rate is given by:

$$\begin{array}{c} \lambda \left(t\right)=\frac{\alpha }{\lambda }{\left[\frac{t}{\lambda }\right]}^{\alpha -1} \end{array}$$ (17)

#### (ii). Baseline Log-logistic distribution

The survival time T has a Log-logistic distribution if lnT has a logistic distribution. The probability density function of a Log-logistic distribution is given by:

$$\begin{array}{c} f\left(t\right)=\frac{\alpha \lambda {\left[\alpha t\right]}^{\lambda -1}}{1+{\left[{\left[\alpha t\right]}^{\lambda }\right]}^{2}},\;\alpha \epsilon R,\lambda >0 \end{array}$$ (18)

The survival function is given as:

$$\begin{array}{c} S\left(t\right)=\frac{1}{1+{\left[\alpha t\right]}^{\lambda }} \end{array}$$ (19)

and the hazard function is given as below:

$$\begin{array}{c} \lambda \left(t\right)=\frac{\alpha \lambda {\left[\alpha t\right]}^{\lambda -1}}{1+{\left[\alpha t\right]}^{\lambda }} \end{array}$$ (20)

### Parameters estimation

Frailty models account for the clustering present in grouped event time data. For a right-censored clustered survival data, the observation for subject *j_ϵ_J*_*i*_ = (1, …, *n*_*i*_) from the cluster *i_ϵ_I* = (1, …, *s*) is the couple (*y*_*ij*_,σ_ij_), where *y*_*ij*_ = *min*(*t*_*ij*_, *c*_*ij*_) is the minimum between the survival time *t*_*ij*_ and the censoring time *c*_*ij*_ and the indicator σ_ij_ = *I*(*t*_*ij*_ ≤ *c*_*ij*_) is one for a subject where the event has occurred, while *σ*=0 for a censored observation. When the covariate information has been collected, the observation will be (*y*_*ij*_, *σ*_*ij*_, *X*_*ij*_), where *X*_*ij*_ denotes the vector of covariates for the *ij*^*th*^ observation. In the parametric setting, the estimation is based on the marginal likelihood in which the frailties have been integrated by averaging the conditional likelihood with respect to the frailty distribution.

Under the assumption of right censoring and of independence between the censoring time and the survival time of random variables, given the covariate information, the marginal log-likelihood of the observed data can be given as:

L_*marg*_(ψ, *β*, *θ*, *Z*, *X*)=

$$\begin{array}{c} \sum_{i=1}^{s}\{[\sum_{j=1}^{n_{i}}\sigma_{ij}\left(log\left(\lambda_{0}\left(y_{ij}\right)\right)+X_{ij}^{T}\beta \right)]+log\left(-1^{\left(d_{i}\right)}\right)L^{\left(d_{i}\right)}([\sum_{j=1}^{n_{i}}H_{0}\left(y_{ij}\right)\text{exp}\left(X_{ij}^{T}\beta \right)])\} \end{array}$$ (21)

Where d_*i*_ = $\sum_{j=1}^{n_{i}}\sigma_{ij}$ is the number of events in the *i*^*th*^ cluster and *L*^(*q*)^(.) is the *q*^*th*^ derivative of the Laplace transform of the frailty distribution Z is defined as:

$$\text{L}\left(\text{s}\right)=\text{E}\left[\text{exp}\left(-Zs\right)\right]=\int_{0}^{\infty }\text{exp}\left(Z_{i}s\right)f\left(z_{ij}\right)dz_{i},s>0$$

$$L^{\left(q\right)}\left(\text{s}\right)={\left(-1\right)}^{q}\int_{0}^{\infty }Z^{q}\text{exp}\left(-Zs\right)f\left(z\right)dz,q>0$$

Where *φ* represents a vector of parameters of the baseline hazard function, *β* is the vector of regression coefficients, and the variance of the random effect. The estimates of *φ*, *β*, *θ* are obtained by maximizing the marginal log-likelihood of the above. This can be done if one is able to compute the higher order derivatives *L*^(*q*)^(.) of the Laplace transform up to *q* = *max*(*d*1, …, *ds*).

#### Test of hetroginity

In frailty models, *θ* is estimated to get an idea on heterogeneity in the outcome among clusters. When *θ* is large and differs significantly from zero; it reflects heterogeneity among clusters and a strong association among individuals in the same cluster. On the other hand, when *θ* is equal to zero, the frailties are identically equal to one which implies that the cluster effects are not present and events are independent within and across centers [27]. The likelihood ratio test is used for comparing the models with and without frailties. In other words, it is used for testing the null hypothesis *H*_*o*_: *θ* = 0 versus the alternative hypothesis *H*_1_: *θ* >0. This heterogeneity parameter *θ* from the frailty models was estimated using the Penalized Partial Likelihood (PPL) technique. Since the null hypothesis is at the boundary of the parameter space, the LR test statistic is not the usual $\chi_{1}^{2}$ but rather is a 50:50 mixture of the chi-square distribution with 0 and 1 degree of freedom, denoted as $\chi_{01}^{2}$ was used, and thus requires careful consideration concerning the calculation of its p-value [22].

### Comparison of the models

Model comparison and selection are among the most common problems of statistical practice, with numerous procedures for choosing among a set of models [28]. There are several methods of model selection. The most commonly used methods include information criteria. One of the most commonly used model selection criteria is Akaike Information Criterion(AIC). A data-driven model selection method such as an adapted version of Akaike’s information criterion AIC [29] is used to find the truncation point of the series. In some circumstances, it might be useful to easily obtain AIC values for a series of candidate models [30]. The model with the smallest AIC value is considered a better fit. For each model, the value is computed as **AIC=-2log(L)+2(k)**

Where: k is the number of parameters and L is the maximized likelihood value. The preferable model is the one with the lowest value of the AIC [30].

### Model diagnostics

#### The Cox-Snell residuals

The Cox-Snell residuals method can be applied to any parametric model and the residual plots can be used to check the goodness of fit. For the parametric regression problem, analogs of the semi-parametric residual plots can be made with a redefinition of the various residuals to incorporate the parametric form of the baseline hazard rates [17]. In general, the Cox-Snell residual provides a check of the overall fit of the model [31].

## Results and discussion

### Results

The descriptive summary of baseline categorical covariates are given in Table 2. The medical cards of 205 leprosy patients were reviewed, 69(33.7%) experienced some disability (G1D or G2D) during treatment.

**Table 2:** Descriptive summary of categorical variables for leprosy patients dataset, LPAC, 2015–2019.

Variables	Category	Number of patients	Event^1^ (%)
Sex	Male	133	56(27.32)
	Female	72	13(6.34)
Region	Addis Ababa	42	2(0.98)
	Amhara	61	28(13.66)
	Oromiya	52	22(10.73)
	SNNP	26	7(3.41)
	Others	24	12(5.85)
Treatment Category	Relapse	86	41(20)
	Defaulter	17	12(5.85)
	New	102	16(7.80)
Starting disability grade	Zero	80	19(9.27)
	One	125	50(24.39)
Leprosy Type	MB	174	67(32.7)
	PB	31	2(0.98)
First Lesion	Hand	83	37(18.05)
	Feet	53	21(10.24)
	Hand and Feet	17	7(3.41)
	Face	52	4(1.95)
Lesion Type	Macules	133	37(18.05)
	Plagues	28	12(5.85)
	Nodules	41	18(8.78)
	Macules and Nodules	3	2(0.98)
Sensation Loss	Moderate	95	51(24.88)
	Marked	27	8(3.90)
	Absent	83	10(4.88)
Lesion Distribution	Part Symmetric	83	21(10.24)
	Symmetric	93	25(12.19)
	Asymmetric	29	23(11.22)
Thickened Nerve	Yes	76	40(19.51)
	No	129	29(14.15)
Exposure History	No	185	57(27.80)
	Contact	11	5(2.44)
	Family	9	7(3.41)
Smear Result	Zero	8	4(1.95)
	Positive	183	59(28.78)
	Negative	14	6(2.93)

^1^ = Some Disability (G1D or G2D) during treatment

Relatively among the region of Ethiopia, leprosy patients from the Amhara region were the highest in the number to experience some disability 28 (13.66%) followed by Oromiya 22 (10.73%). Addis Ababa city administration was the one that relatively lowest in the number of leprosy patients who experience some disability during treatment 2 (0.98%), followed by SNNPR 7 (3.41%). Patients who were male 56(27.32%) experienced more of some disability than females 13(6.34%). Out of the total leprosy patients included in the study, patients who have no family history of the disease or any contact with a leprosy patient were the highest in number to have some disability during treatment 57(27.8%) compared to other exposure history of patients. These were followed by leprosy patients who had a family history of the disease with 3.41%. The category of exposure history of leprosy patients with the least percentage of experiencing some disability was those who had contact with a leprosy patient with 2.44%.

In this study, leprosy patients with thickened nerves recorded the highest percentage of some disability 19.51% during treatment as compared to those without thickened nerves 14.15%. Most of the patients in this study were to have MB. In this study, 174 leprosy patients were with MB and they also recorded the highest percentage of some disability 32.7% as compared to those with PB which was recorded to be 0.98%. 59(28.78%) of some disability was prevalent among the patients having positive smear results. About 37(18.05%) of some disabilities were attributed to leprosy patients who have a lesion on their hands at the first time of the disease. Most of the patients in this study were to have a symmetrical lesion distribution on their body, and they also recorded the highest percentage of some disability 25(12.19%) as compared to those whose lesions distributed as asymmetric 23(11.22%) of some disability and part symmetric 21(10.24%).

The mean age of leprosy patients at enrollment for the treatment was 33.78 years with a standard deviation (Std.dev) of 14.63, the minimum and maximum age in years were 6 and 80 respectively. The mean duration of symptoms for patients at baseline with standard deviation (Std.dev) was 29.21 (22.53) months and the minimum and maximum duration were 1 and 96 months respectively (Table 3).

**Table 3:** Baseline characteristics of continuous variables, LPAC, 2015–2019.

variables	N	Minimum	Maximum	Mean	Std. dev.*
Age in years	205	6	80	33.78	14.63
Symptom Duration	205	1	96	29.21	22.53

*=standard deviation

#### Non-parametric exploratory analysis methods

**Fig 1:**
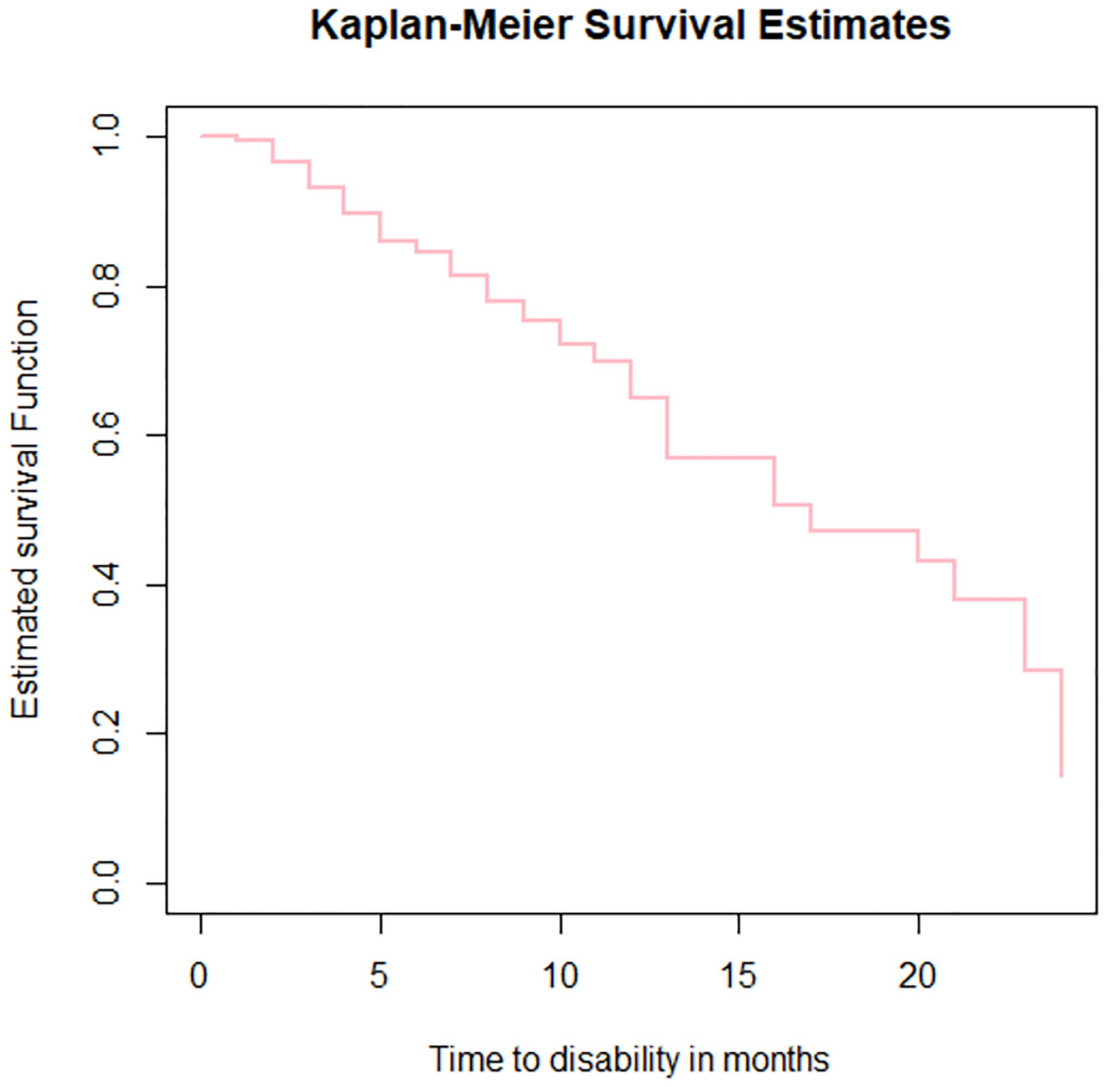
The K-M plot of the survival function for leprosy patients dataset, LPAC, 2015–2019.

*The Kaplan- Meier Estimate*. The Kaplan-Meier plot of the survival function for leprosy patients dataset is shown below (Fig 1).

**Fig 2:**
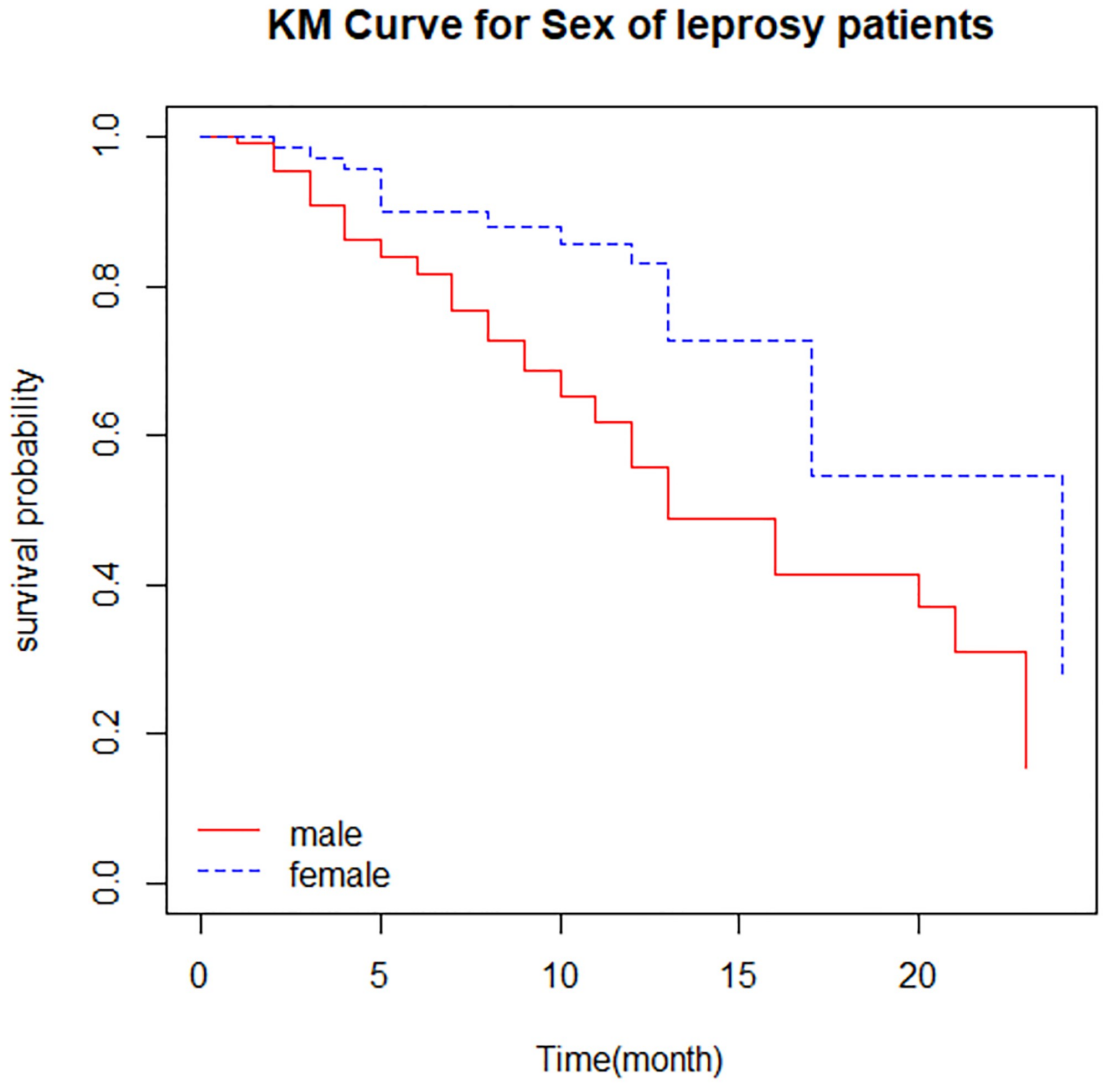
The survival function by sex of leprosy patients.

**Fig 3:**
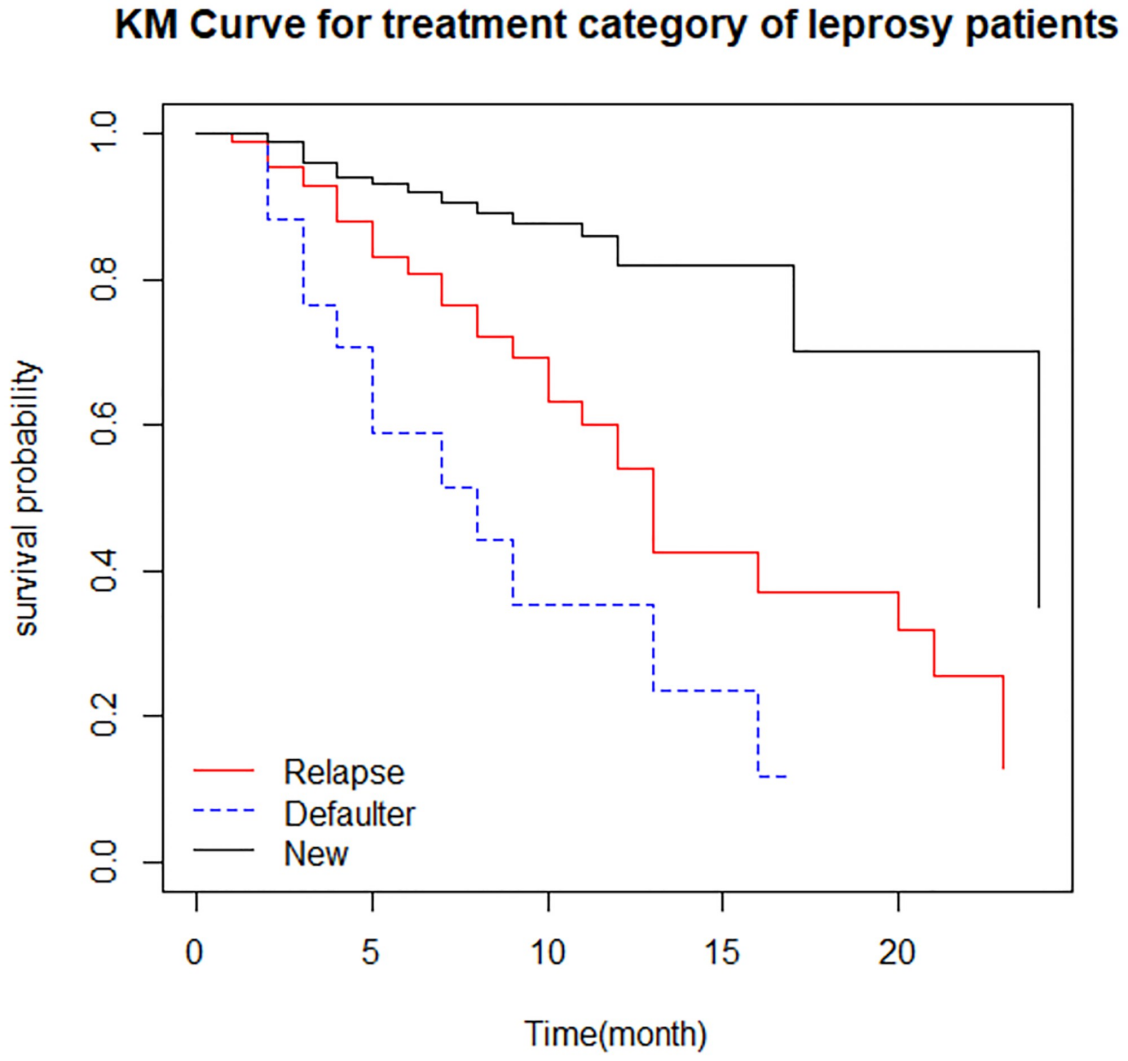
The survival function by treatment category of leprosy patients.

*Survival function of different groups*. There is a higher survival experience for a patient who were female (Fig 2) and newer to the treatment (Fig 3).

*Comparison of survival time*. The log-rank test statistics were used to test whether there is a difference or not in the survival curve between two or more groups. The log-rank test revealed that there is no difference in the survival function of patient lesions distribution, lesion type, smear result, disability grade, leprosy type, and exposure history since their corresponding p-value is greater than the common alpha level of significance. However, in the other independent variables, there was a significant variation in the survival function of different categories (P-value <0.05) (Table 4).

**Table 4:** Results of the log-rank test for each categorical variable, LPAC, 2015–2019.

Categorical variables	DF*	Chi-squ	P-value
Sex of patient	1	10	.002
Treatment Category	2	20.4	<.0001
First Lesion	3	213	.005
Sensation Loss	2	26.9	<.0001
Lesion Distribution	2	31.5	0.17
Thickened Nerve	1	8	.005
Lesion type	3	6.3	0.1
Smear Result	2	3.4	0.2
Disability grade	1	0	0.9
Leprosy type	1	3.6	0.06
Exposure History	2	2.5	0.3

*=Degree of Freedom

#### Accelerated failure time model results

*Univariable analysis*. Univariable analysis was performed in order to see the effect of each covariate and to select variables to be included in the multivariable analysis. The univariable analysis was fitted for every covariate by using two baseline distributions i.e. Weibull and log-logistic. In both univariable analysis models, sex of the patient, age, treatment category, symptom duration, first lesion body part, sensory loss, and thickened nerve were significantly associated with disability status at 20% level of significance. Those variables are candidate variables for multivariable analysis.

*Multivariable analysis for accelerated failure time analysis*. For disability data, Multivariable Accelerated Failure Time(AFT) model with the two baseline distributions Weibull and loglogistic were fitted by including all the covariates that were significant in the univariable analysis. Accordingly, the Log-logistic Accelerated Failure Time model (AIC = 519) was found to be the best for the disability the from leprosy data set since it has a smaller AIC value (Table 5).

**Table 5:** Comparison of AFT models using AIC criteria for leprosy patients at ALERT (LPAC, From 2015–2019).

Baseline Distribution	AIC*
Weibull	525
**Log-logistic**	**519**

*=Akaikei Information Criteria

The results log-logistic multivariable AFT model showed that the predictor covariates Sex of patient, Age, Treatment Category, Symptom duration, and Sensory Loss were significantly associated with some disability status at 5% level of significance. The remaining variables which were used in the univariable analysis were found to be non-significant (Table 6).

**Table 6:** Summary result for log-logistic acceleration failure time model for leprosy patients at ALERT center(LPAC, from 2015–2019).

variables	Category	Estimate(*β*)	SE(*β*)	Φ	95%CI for Φ	P-Value
Sex of Patient	Male(1)				…	
	Female	0.418	0.210	1.519	[1.006, 2.293]	0.047
Age		-0.019	0.006	0.981	[0.969, 0.993]	0.002
Category	Relapse(1)				…	
	Defaulter	-0.711	0.249	0.491	[0.301, 0.801]	0.004
	New	0.566	0.197	1.761	[1.196, 2.594]	0.004
Duration		-0.011	0.004	0.989	[0.981, 0.997]	0.008
First Lesion	Hand(1)				…	
	Leg	-0.134	0.196	0.875	[0.596, 1.284]	0.494
	HL	0.494	0.302	1.639	[0.906, 2.963]	0.102
	Face	0.090	0.301	1.094	[0.607 1.973]	0.764
Sensory Loss	Marked(1)				…	
	Moderate	0.429	0.260	1.537	[0.923, 2.559]	0.099
	Absent	0.822	0.220	2.276	[1.478, 3.506]	0.000
Thickened	Yes(1)				…	
	No	-0.065	0.185	0.937	[0.653, 1.346]	0.726
Scale = 0.467						
shape = 2.14						

SE(*β*): standarderror for *β*,

1 = Reference

Φ = Acceleration factor

*Tests of unobserved heterogeneity and model comparisons*. The variance of the frailty was significant for all shared frailty distribution with log-logistic baseline hazard function, whereas it was not significant in the weibull baseline hazard function using the same shared frailty distribution at 5% level of significance. Moreover, the AIC value was higher for the Weibull baseline hazard function. Parametric shared frailty analysis was done by log-logistic baseline hazard function for gamma, Inverse-Gaussian, and Positive Stable shared frailty distributions. The heterogeneity parameter (variance of the random term) for log-logistic-gamma, log-logistic -Inverse Gaussian and log-logistic-Positive Stable models were 0.848, 0.935 and 0.451 with p-values 0.001, 0.004 and 0.02 respectively. Thus, from these results, we can conclude that unobservable heterogeneity is significant at the 5% level of significance. Among those models, Gamma shared frailty model with the log-logistic baseline hazard function has the smallest AIC (517). This indicates that under the given scenario, it is relatively the most appropriate model to describe disability data (Table 7).

**Table 7:** The value of AIC for parametric shared frailty models, LPAC, 2015–2019.

Baseline	shared frailty	AIC^1^
Log-logistic	Gamma	**517**
	Positive Stable	523
	Inverse-Gaussian	520
Weibull	Gamma	555
	Positive Stable	529
	Inverse-Gaussian	543

^1^ = Akaikei Information Criteria

The effect of random component (frailty) was significant for all shared frailty models and log-logistic gamma shared frailty model has minimum Akaike information criteria (AIC = 517). This indicates the Log-logistic-gamma shared frailty model is a more efficient model to describe disability from the leprosy dataset. The frailty term in this model is assumed to follow a gamma distribution with mean 1 and variance equal to theta (*θ*). The estimated value of theta (*θ*) is 0.848. A likelihood ratio test for the hypothesis *θ*= 0 was a significant P-value of 0.001. This implied that the frailty component had a significant contribution to the model. And the associated Kendall’s tau (*τ*), which measures dependence within clusters (regions), is estimated to be 0.298 (Table 8).

**Table 8:** Results of the final log-logistic-gamma shared frailty model, LPAC, 2015–2019.

variables	Category	Estimate(*β*)	SE(*β*)	HR	95%CI for HR	P-Value
Sex of Patient	Male(1)				…	
	Female	-0.341	0.352	0.711	[0.357, 1.418]	0.333
Age		0.033	0.011	1.033	[1.011, 1.055]	0.003 **
Category	Relapse(1)				…	
	Defaulter	1.003	0.386	2.726	[1.280, 5.806]	0.009 **
	New	-1.111	0.329	0.329	[0.173, 0.628]	0.001 ***
Duration		0.013	0.006	1.013	[1.001, 1.026]	0.036 *
First Lesion	Hand(1)				…	
	Leg	0.433	0.301	1.542	[0.855, 2.783]	0.15
	HL	-0.467	0.442	0.627	[0.264, 1.491]	0.291
	Face	-0.035	0.587	0.966	[0.305, 3.052]	0.952
Sensory Loss	Marked(1)				…	
	Moderate	-0.056	0.440	0.946	[0.399, 2.240]	0.899
	Absent	-1.019	0.374	0.361	[0.173, 0.751]	0.006 **
Thickened	Yes(1)				…	
	No	0.126	0.307	1.134	[0.621, 2.071]	0.682
Frailty Term	*θ*	0.848				0.0010
	*τ*	0.298				
Scale = 1.72						
shape =-6.34						

SE(*β*): standarderror for *β*,

1 = Reference

* = significant

The confidence intervals of the HR for all significant covariates do not include one at the 5% level of significance. This showed that they are significant factors for determining some disability among leprosy patients in ALERT Hospital Addis Ababa, Ethiopia. The HR of the age of patients with leprosy was about 1.033 implying that for a unit increases in age, the hazard for the occurrence of some disability was significantly increased by 3.3% (p-value = 0.003) keeping all variables constant. The HR for patients who were defaulters was 2.726 [1.280, 5.806] times greater and reduced the risk of experiencing some disability by 33% for patients who were new for treatment as compared with patients in the relapse category. Duration of the disease before starting the treatment for a patient was another factor that significantly predicts having some disability during treatment. The hazard ratio for the disease duration of a patient was (HR = 1.013). This implies, that the hazard of experiencing some disability was significantly increased by 1.3% (p-value = 0.036) for a unit increase in the duration of disease before treatment, keeping all variables constant. In addition, there was a reduced risk of experiencing some disability by 36% for patients who do not have a sensory loss at diagnosis. The HR was 0.361[0.173, 0.751]and since the confidence interval did not include one and the p-value was very small (P = 0.006) indicating that loss of sensation was a significant factor to determine the disability from leprosy at 5% level of significance (Table 8).

### Model diagnostics

**Fig 4:**
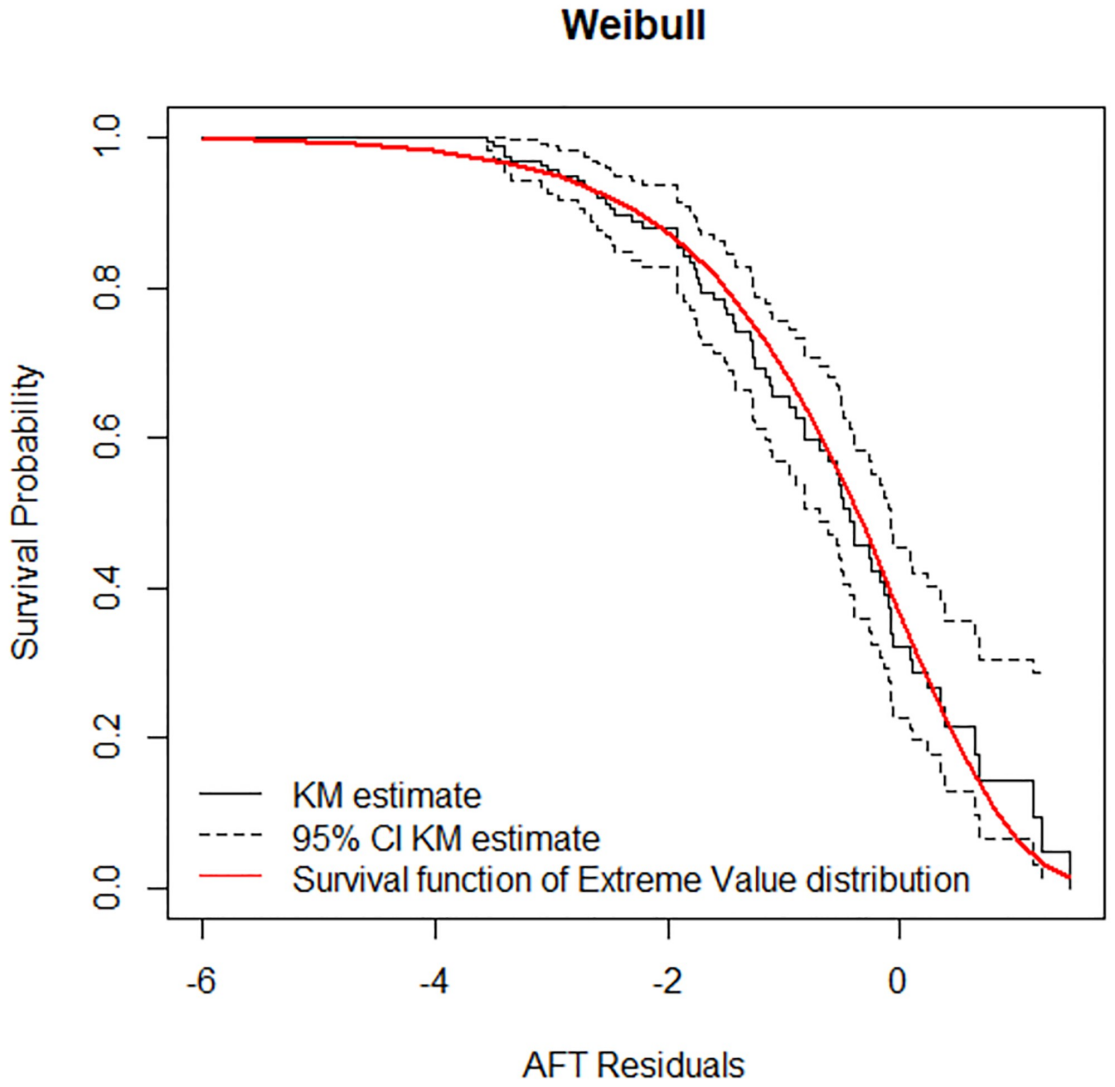
Estimated cumulative hazard plot of the Cox-Snell residuals for the Weibull baseline distribution, LPAC, 2015–2019.

**Fig 5:**
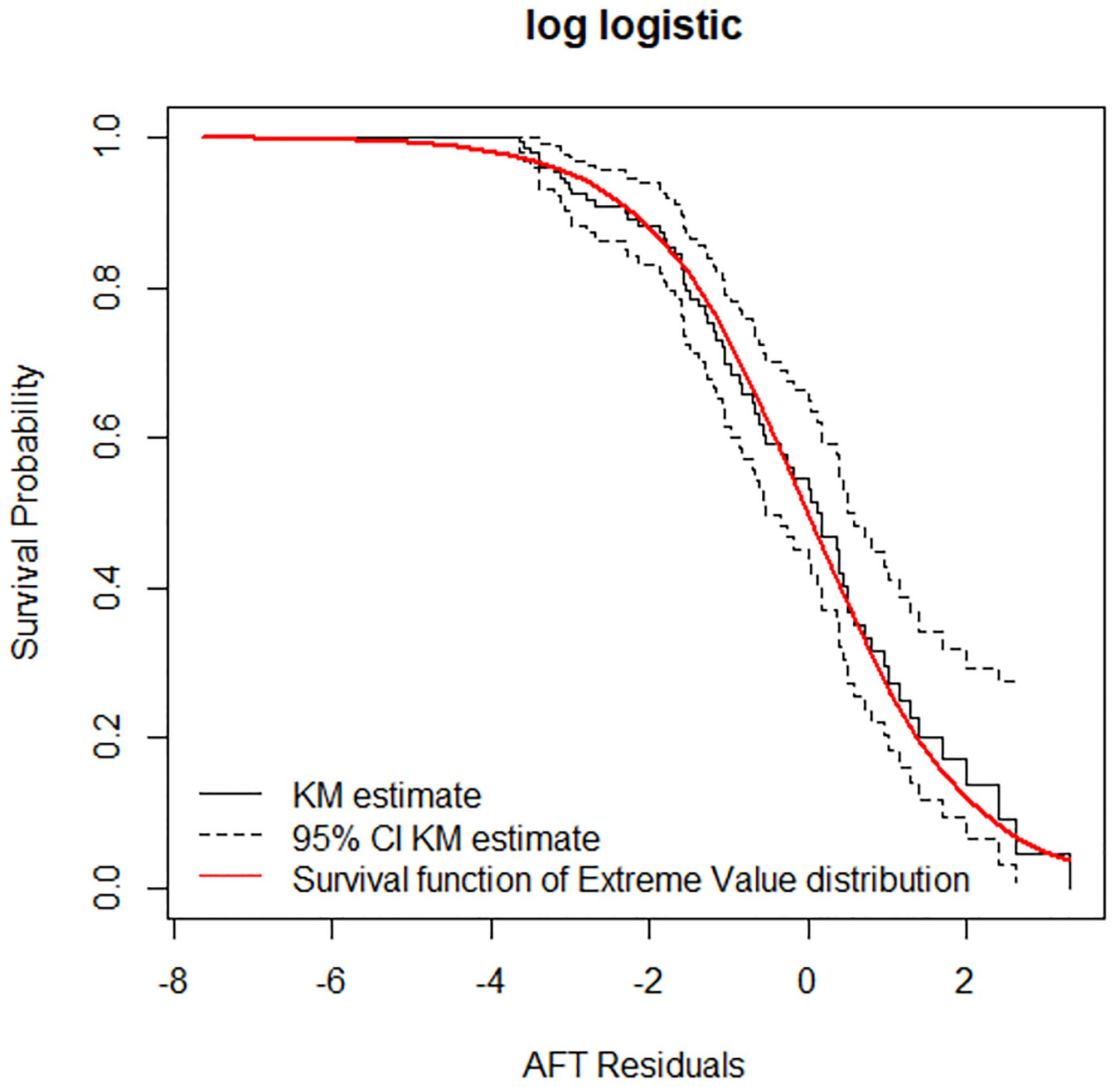
Estimated cumulative hazard plot of the Cox-Snell residuals for the log-logistic baseline distribution, LPAC, 2015–2019.

The Cox-Snell residuals had been obtained from fitting the Weibull and log logistic models to the data. The figures below display the diagnostic based on Cox-Snell residuals with the 95% point wise CI for the Kaplan-Meier estimate of the Cox-Snell residuals along the red line. Since the Weibull distribution becomes to be below the lower confidence interval at the end (Fig 4), but for log-logistic, the line is more in touch with the Kaplan-Meier estimate line and completely within the confidence intervals therefore the log-logistic distribution provides a good fit to the data (Fig 5).

#### The Cox Snell residual plots

**Fig 6:**
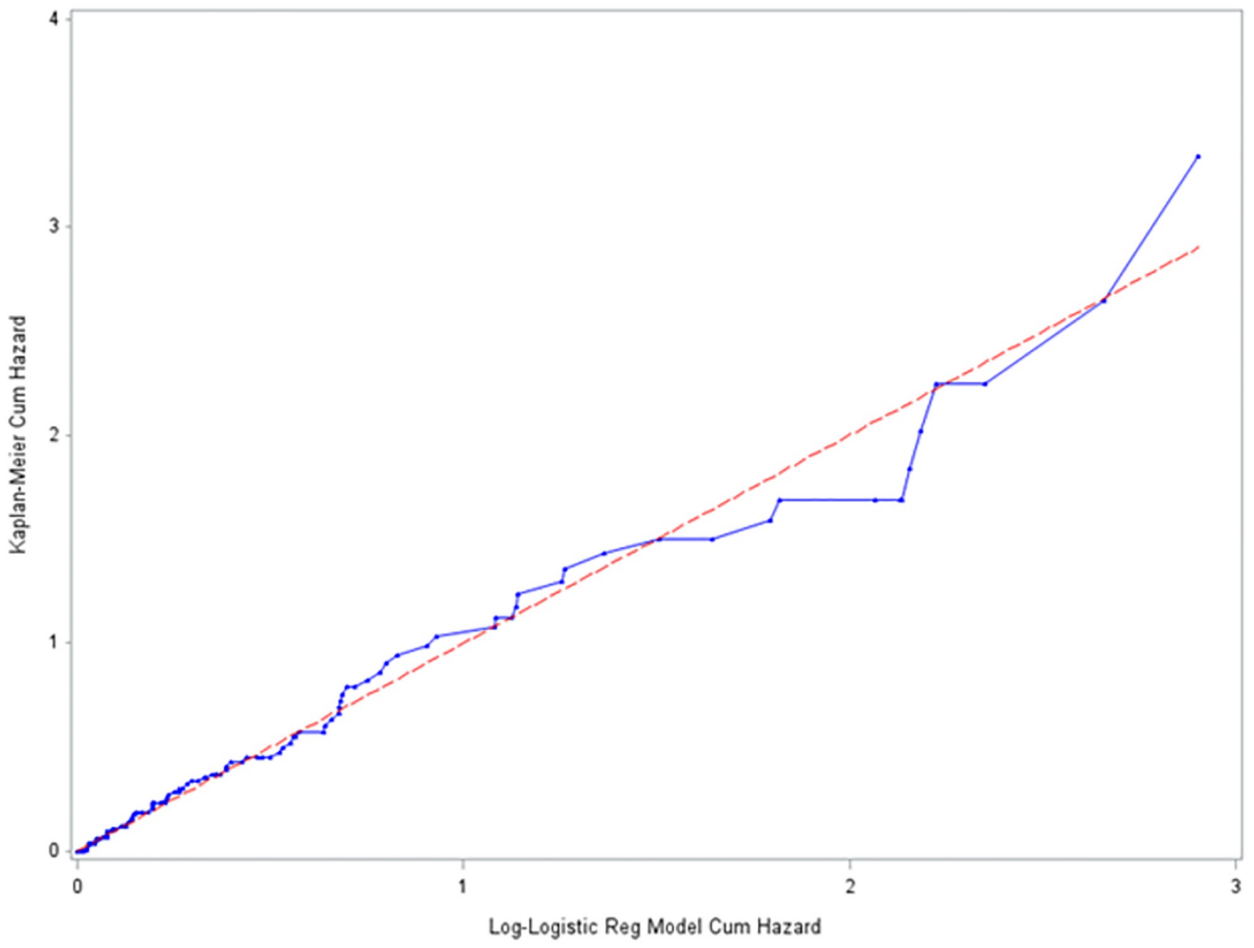
Estimated cumulative hazard plot of the Cox-Snell residuals for the log-logistic baseline distribution, LPAC, 2015–2019.

**Fig 7:**
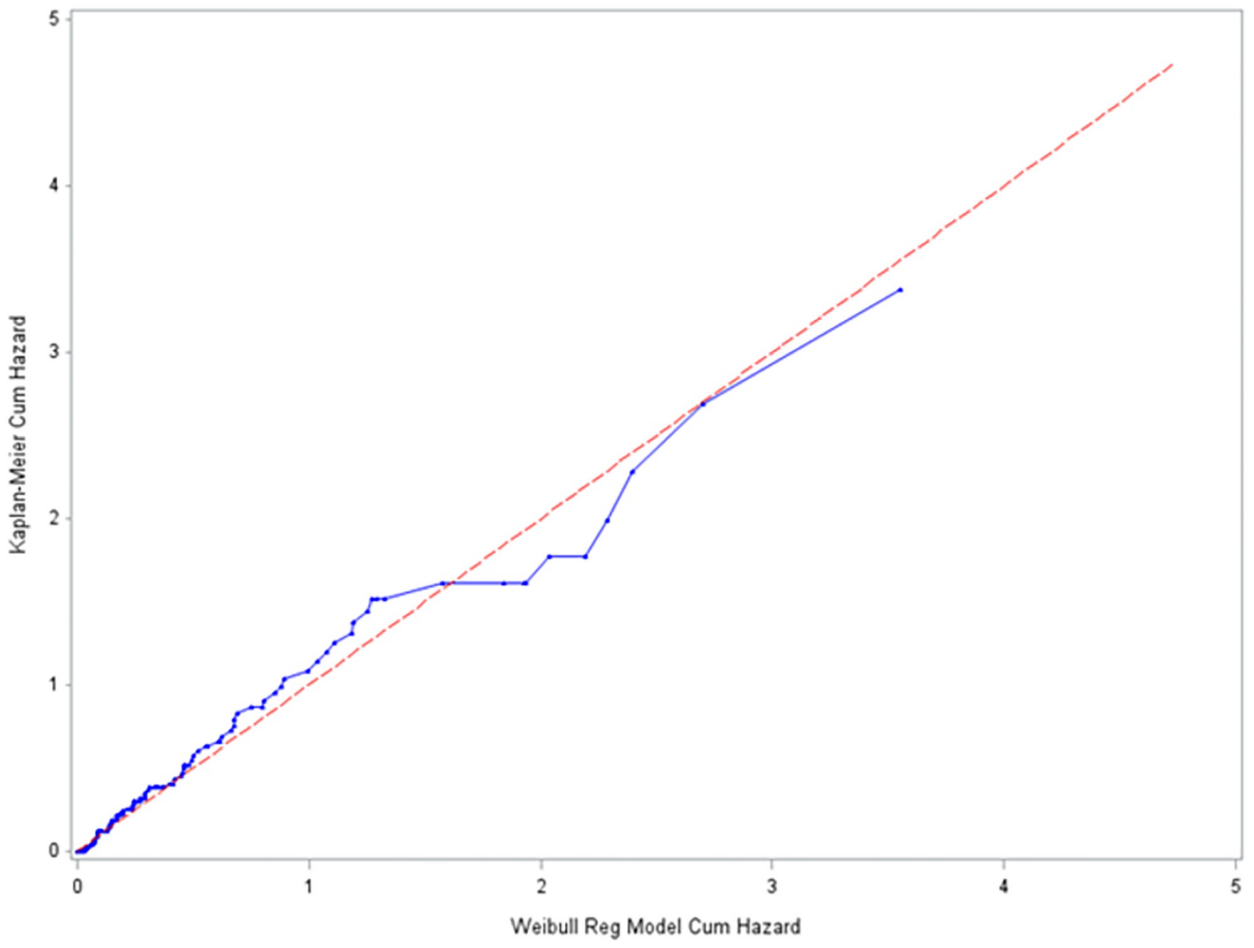
Estimated cumulative hazard plot of the Cox-Snell residuals for the Weibull baseline distribution, LPAC, 2015–2019.

The Cox-Snell residuals (together with their cumulative hazard function) had been obtained by fitting the Weibull and the log-logistic models to the data. It can be seen that the plot of the cumulative hazard function against Cox-Snell residuals is close to the straight lines up to the end for the log-logistic model (Fig 6) when compared to the Weibull model (Fig 7). This suggested that log-logistic provided the best fit for the determinants of disability among leprosy patients in the ALERT center in Addis Ababa Ethiopia.

#### Checking for overall goodness of fit

**Fig 8:**
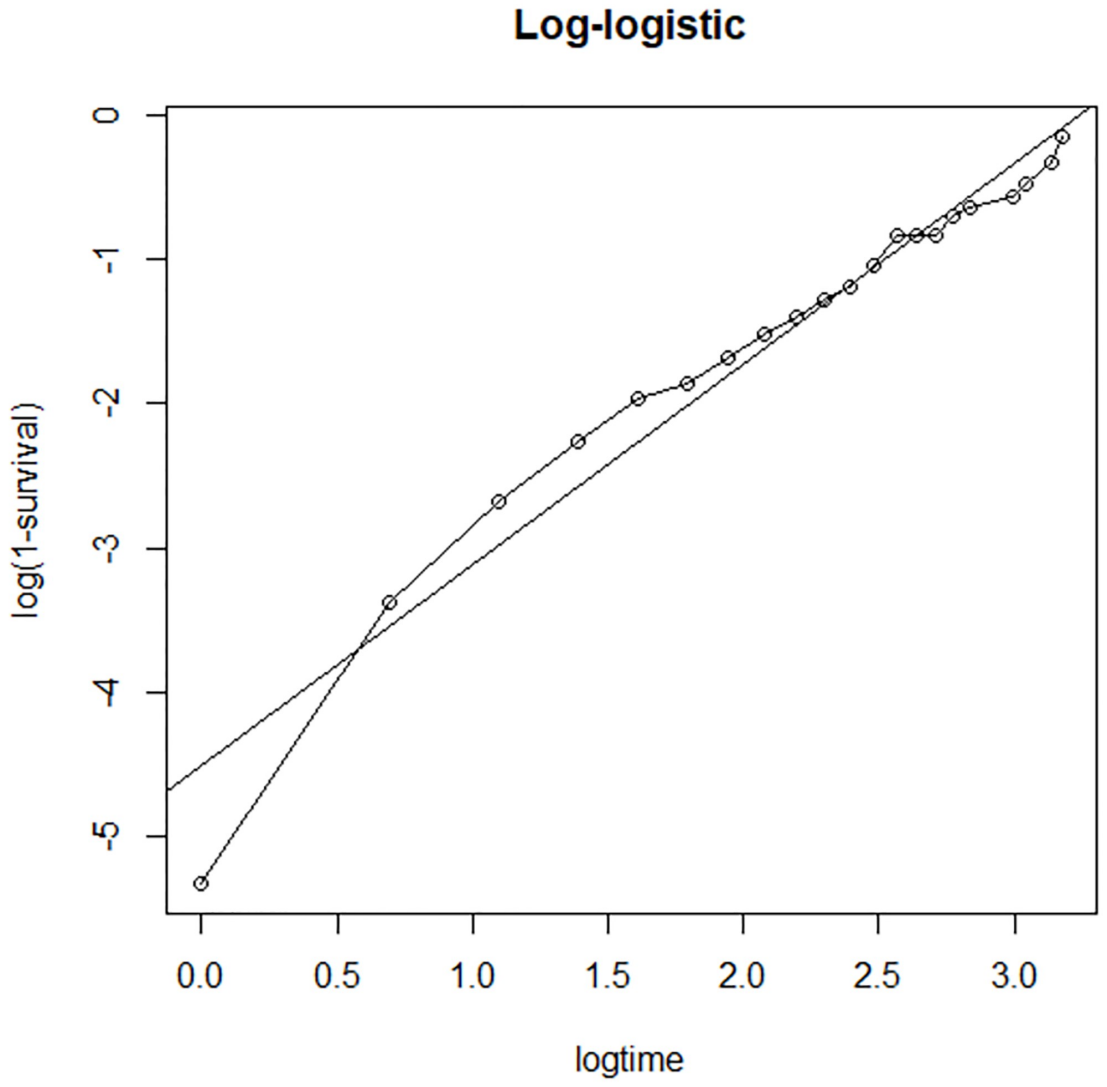
Adequacy checking for the parametric log-logistic baseline distribution, LPAC, 2015–2019.

**Fig 9:**
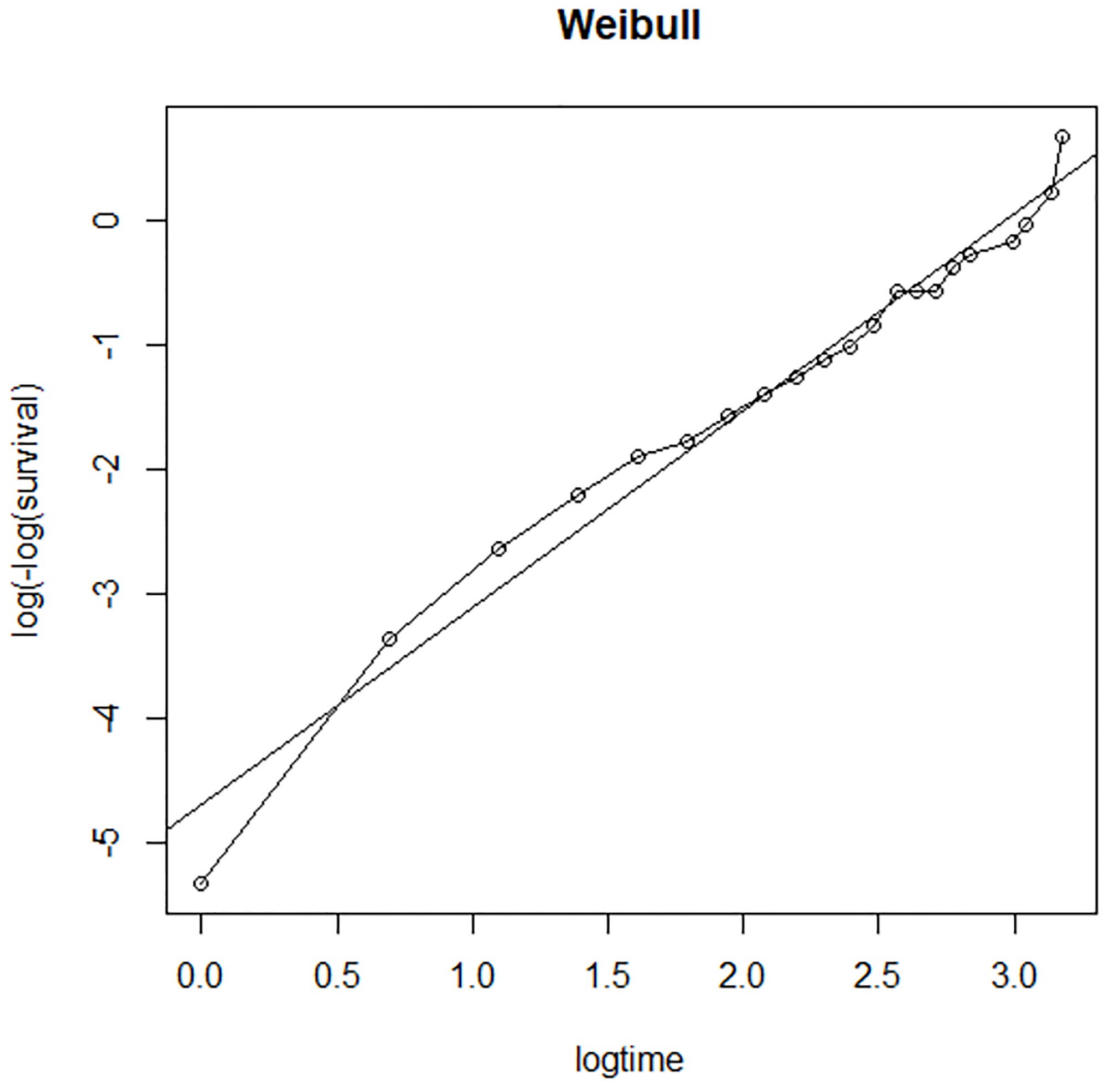
Adequacy checking for the parametric Weibull baseline distribution, LPAC, 2015–2019.

The final step in the model assessment is to see the overall goodness of fit. Therefore, it is desirable to determine whether a fitted parametric model adequately describes the data or not. From the two plots, it seems that Log-logistic is more linear (Fig 8) than the Weibull distribution (Fig 9). This indicates that Log-logistic is more appropriate.

### Discussion

The findings of this study revealed that there is heterogeneity between patients categorized as regions and correlation within the same region of leprosy patients. In all shared frailty models with a log-logistic baseline, the variance of the random term is statistically significant at 5% level of significance. This showed that we have to include the effect of random terms in our models since the unobserved heterogeneity within regions cannot be ignored.

There are three types of frailty models: shared, nested, and joint frailty models. For the comparison of distributions of the models, AIC is used for the shared frailty model while LRT is used for nested and joint frailty models [32]. Therefore, for this study, the comparison was done by using the AIC criteria, where a model with minimum AIC is accepted to be the best [30]. Accordingly, log-logistic-Gamma shared frailty model which has AIC value of 517 was the most appropriate model. According to the findings in the preceding result, this study attains to the following discussions.

Based on the given dataset age of the patient was the first factor with disability due to leprosy as was indicated in log-logistic-gamma shared frailty model. The HR of the age of patients with leprosy in the log-logistic-gamma shared frailty models was about 1.033 implying that for a unit increased in age, the hazard of disability of patients was significantly increased by 3.3% (p-value = 0.003) keeping all variables constant. This result is similar to a study done by [33] that shows for patients in the age group greater or equal to 30, disability was high; as people in this age group are engaged in physical work, they are vulnerable to different kinds of injuries. Our study also confirms a lower chance of disability for patients who have no sensory lose. The risk of development of disability was reduced in those patients who do not loss sensory. Sensory function loss is a cause of repeated injury, ulceration, and limb shortening. Corneal sensation loss may result in unrecognized corneal injury and significant visual loss. Motor function loss is a cause of finger and toe clawing, failure of eye closure (lagophthalmos), and foot and wrist drop. This is also consistent with the results of a previous study by [33] in Ethiopia reported that those who presented with sensory loss were also more likely to have a disability.

Although we noticed an increased occurrence of disability in patients seeking treatment late after the onset of the disease (long symptom duration), A long duration of symptoms for leprosy patients before seeking treatment has been significant risk factor for disability among leprosy cases. This may arise as patients ignored the initial symptoms as they thought the symptoms would disappear by themselves. Most studies have reported a statistically significant association between delay in diagnosis or a long duration of disease and Grade 2 disability. The results of this study found that the hazard of disability for patients was significantly increased by 1.3%(HR = 1.013) for a unit increase in the duration of disease before treatment. This finding is consistent with studies on Prevalence of Disability and Associated Factors among Registered Leprosy Patients in All Africa TB and Leprosy Rehabilitation and Training Centre. The study shows that the longer duration of symptoms leads to disability [33]. This result was also in agreement with the findings in India by [8] which notes that when the patient delay was more than three months, the odds of G2D/G1D at the time of diagnosis were significantly higher (Adj OR = 1.6, 95% CI: 1.3–2.2) among respondents compared to those with patient’s delay of fewer than three months. This is also consistent with the results of previous studies which showed a higher risk of disability in patients who had a symptoms for 6 to 12 months and more than 24 months [2].

There is an increased occurrence of disability in patients with nerve thickening, especially multiple nerve trunk enlargement was associated with a higher chance for disability, this was not found to be statistically significant. Most studies have reported a statistically significant association between nerve thickening and disability [34]. This discordance in our study could be explained by the patient profile disparity at the starting of the treatment and the onset of follow-up as patients may not develop multiple nerve trunk enlargement at the beginning and this nerve thickening might be more in patients with complex progression of the disease. Even if in this study the sex and first lesion body part were not found to be statistically significant, the higher rate of disability associated with the male sex in our study was noted by others as well [35]. It can be explained by the fact that males are more prone to trauma to hands and feet as part of their occupation. [36] described feet as the most common site of disability, while others including us noted hands to be the most commonly involved site as far as disability was concerned [37]. Another potential risk factor for disability was the treatment category of patients. Log-logistic-gamma shared frailty model showed that the treatment category was significantly associated with disability due to leprosy, which was inconsistent with the results by [33].

## Conclusion

This study was based on a dataset of leprosy patients obtained from All Africa TB and Leprosy Rehabilitation and Training Center with the aim of assessing the determinants of leprosy disability by using different parametric baselines with different shared frailty models. Out of the total 205 leprosy patients, 69(33.7%) were experienced the event or disabled during treatment. Using AIC, the log-logistic-Gamma shared frailty model is better fitted to the dataset than other parametric shared frailty models. There is a frailty (clustering) effect on the leprosy patient’s dataset that arises due to differences in the distribution of time to disability among regions. The results of the log-logistic-Gamma shared frailty model showed that the factors that determine the timing of disability are the age of patients, the duration of disease before starting the treatment (symptom duration), treatment category of leprosy patient, and sensory loss are statistically significant. In general, the risk of disability was higher as age increased, for patients with a long duration of symptoms, for defaulter patients and the risk of disability was lower for patients who do not lose their sensation. This study also found the existence of a difference in the patient’s survival curves between two or more groups of covariates. The results indicate that there is a higher survival experience for patients who were female, newer for the treatment, and for patients who don’t lose sensation.

To reduce the patient-related delay (long duration), the program should lay greater emphasis on raising awareness of the community focusing on key messages like symptoms, disability consequence of late detection, availability of free treatment, and availability of leprosy care in a public health facility. Additionally, programs should put more emphasis on early case detection campaigns like an active surveys. Furthermore, media have to work with some proven strategies like: Stigma Elimination Program and in reducing the burden due to physical, mental, and socioeconomic consequences of leprosy on persons affected and their families.

## Supporting information

S1 Data (CSV)

## List of abbreviations

AFT: Accelerated Failure Time
AHRI: Armauher Hansen Research Institute.
AIC: Akaki Information Criterion
ALERT: All Africa Leprosy, Tuberculosis and Rehabilitation Training Centre
BC: Before Christ
DG: Disability Degree
ENAPAL: Ethiopian National Association of Peoples Affected by Leprosy
FMOH: Federal Ministry of Health
G0D: Grade Zero Disability
G1D: Grade One Disability
G2D: Grade Two Disability
GLRI: German Leprosy and TB Relief Association
HR: Hazard Ratio
ILEP: International Federation of Anti-Leprosy Associations
LPAC: Leprosy Patients ALERT Center
LR: Likelihood Ratio
MB: Multi-bacillary
MDT: Multidrug therapy
PAL: Peoples Affected by Leprosy
PB: Pauci-bacillary
PPL: Penalized Partial Likelihood
SNNPR: Southern Nations, Nationalities, and Peoples’ Region
TB: Tuberculosis
WHO: World Health Organization
